# Reply to “Predictors of Recurrence for T3a RCC: A Recurring Conundrum”

**DOI:** 10.3390/diagnostics10110984

**Published:** 2020-11-21

**Authors:** Makito Miyake, Takuto Shimizu, Shunta Hori, Kota Iida, Yasushi Nakai, Kiyohide Fujimoto

**Affiliations:** Department of Urology, Nara Medical University, 840 Shijo-cho, Kashihara, Nara 634-8522, Japan; takutea19@gmail.com (T.S.); horimaus@gmail.com (S.H.); kota1006ida@yahoo.co.jp (K.I.); nakaiyasusiuro@live.jp (Y.N.); kiyokun@naramed-u.ac.jp (K.F.)

We really appreciate Leopold et al’s thoughtful comments in response to our manuscript regarding the prognostic value of pT3 renal cell carcinoma (RCC)-related features. It is our pleasure to discuss this topic in greater depth. The current staging system for T category in renal cell carcinoma (RCC) distinguishes pT1a (≤4 cm), pT1b (4 cm < and ≤ 7 cm), pT2a (7 cm < and ≤ 10 cm), and pT2b (>10 cm) only by tumor diameter. Contrarily, pT3 is not defined by size, but by pathological tumor extension such as perirenal fat invasion (PFI), sinus fat invasion (SFI), or involvement of segmental vessels/main renal vein/IVC (RVI) [1]. Our hypothesis was that three pathological features have similar prognostic value on recurrence rate. With a retrospective review of 91 patients with pT3aN0M0 RCC who underwent radical surgery, patients with tumor size >7 cm, urinary collecting system invasion (UCSI), three invasive sites (PFI + SFI + RVI), and clinically detected renal vein thrombus showed a significant correlation with high recurrence rates after surgery [2]. The multivariate analysis revealed that tumor size of >7 cm, the presence of UCSI, and clinically detected renal vein thrombus were the independent predictors of recurrence. We concluded that tumor size, UCSI, and clinically detected renal vein thrombus should be considered for inclusion in the TNM classification of stage pT3a. Although many previous works have addressed the invasive sites and its effect on prognosis, the results are not consistent. As Leopold et al. commented here, future additional, larger-cohort studies are required to validate the previous evidence and establish a more accurate staging system. The efforts will enable the planning of close follow-up regimens and appropriate early interventions such as neoadjuvant and adjuvant therapy, ultimately leading to improved survival.
